# Upregulation of Ca^2+^-binding proteins contributes to VTA dopamine neuron survival in the early phases of Alzheimer’s disease in Tg2576 mice

**DOI:** 10.1186/s13024-022-00580-6

**Published:** 2022-11-25

**Authors:** Livia La Barbera, Annalisa Nobili, Emma Cauzzi, Ilaria Paoletti, Mauro Federici, Luana Saba, Cecilia Giacomet, Ramona Marino, Paraskevi Krashia, Marcello Melone, Flavio Keller, Nicola Biagio Mercuri, Maria Teresa Viscomi, Fiorenzo Conti, Marcello D’Amelio

**Affiliations:** 1 Department of Medicine and Surgery, Università Campus Bio-Medico di Roma, 00128 Rome, Italy; 2 Department of Experimental Neurosciences, IRCCS Santa Lucia Foundation, 00143 Rome, Italy; 3 Department of Systems Medicine, University of Rome Tor Vergata, 00133 Rome, Italy; 4 Department of Sciences and Technologies for Humans and Environment, Università Campus Bio-Medico di Roma, 00128 Rome, Italy; 5 Section of Neuroscience and Cell Biology, Department of Experimental and Clinical Medicine, Università Politecnica delle Marche (UNIVPM), 60020 Ancona, Italy; 6 Center for Neurobiology of Aging, IRCCS Istituto Nazionale Ricovero e Cura Anziani (INRCA), 60020 Ancona, Italy; 7 Department of Life Science and Public Health; Section of Histology and Embryology, Università Cattolica del Sacro Cuore, 00168 Rome, Italy; 8 Fondazione Policlinico Universitario “A. Gemelli”, IRCCS, 00168 Rome, Italy; 9 Foundation for Molecular Medicine, Università Politecnica delle Marche, 60020 Ancona, Italy

**Keywords:** Calbindin, Calretinin, Autophagy, Mitophagy, Oxidative stress, Calcium, Apoptosis inducing factor, Midbrain, Neurodegeneration, Cell death

## Abstract

**Background:**

Recent clinical and experimental studies have highlighted the involvement of Ventral Tegmental Area (VTA) dopamine (DA) neurons for the early pathogenesis of Alzheimer’s Disease (AD). We have previously described a progressive and selective degeneration of these neurons in the Tg2576 mouse model of AD, long before amyloid-beta plaque formation. The degenerative process in DA neurons is associated with an autophagy flux impairment, whose rescue can prevent neuronal loss. Impairments in autophagy can be the basis for accumulation of damaged mitochondria, leading to disturbance in calcium (Ca^2+^) homeostasis, and to functional and structural deterioration of DA neurons.

**Methods:**

In Tg2576 mice, we performed amperometric recordings of DA levels and analysis of dopaminergic fibers in the Nucleus Accumbens – a major component of the ventral striatum precociously affected in AD patients – together with retrograde tracing, to identify the most vulnerable DA neuron subpopulations in the VTA. Then, we focused on these neurons to analyze mitochondrial integrity and Apoptosis-inducing factor (AIF) localization by electron and confocal microscopy, respectively. Stereological cell count was also used to evaluate degeneration of DA neuron subpopulations containing the Ca^2+^-binding proteins Calbindin-D28K and Calretinin. The expression levels for these proteins were analyzed by western blot and confocal microscopy. Lastly, using electrophysiology and microfluorometry we analyzed VTA DA neuron intrinsic properties and cytosolic free Ca^2+^ levels.

**Results:**

We found a progressive degeneration of mesolimbic DA neurons projecting to the ventral striatum, located in the paranigral nucleus and parabrachial pigmented subnucleus of the VTA. At the onset of degeneration (3 months of age), the vulnerable DA neurons in the Tg2576 accumulate damaged mitochondria, while AIF translocates from the mitochondria to the nucleus. Although we describe an age-dependent loss of the DA neurons expressing Calbindin-D28K or Calretinin, we observed that the remaining cells upregulate the levels of Ca^2+^-binding proteins, and the free cytosolic levels of Ca^2+^ in these neurons are significantly decreased. Coherently, TUNEL-stained Tg2576 DA neurons express lower levels of Calbindin-D28K when compared with non-apoptotic cells.

**Conclusion:**

Overall, our results suggest that the overexpression of Ca^2+^-binding proteins in VTA DA neurons might be an attempt of cells to survive by increasing their ability to buffer free Ca^2+^. Exploring strategies to overexpress Ca^2+^-binding proteins could be fundamental to reduce neuronal suffering and improve cognitive and non-cognitive functions in AD.

**Supplementary Information:**

The online version contains supplementary material available at 10.1186/s13024-022-00580-6.

## Background

Alzheimer’s Disease (AD) is the most common form of dementia in the world, with more than 50 million people affected, causing a huge burden on patients and healthcare costs. Extracellular amyloid beta (Aβ) plaques and intracellular neurofibrillary tau tangles are the main histopathological features of AD, clinically characterized by progressive deterioration of memory and cognition and, ultimately, brain atrophy [1, 2]. AD is also associated with several comorbidities, including neuropsychiatric symptoms like agitation, apathy, depression, anxiety, aggression, and sleep- and night-time disturbances, observed in more than 50% of patients [3–5].

Even though the research has led to important progress in understanding the pathophysiology of AD, the exact molecular and cellular mechanisms underlying the disease still remain unknown and the therapeutic strategies targeting Aβ and tau pathways have largely failed or still remain controversial [6–16]. Thus, in the last years the research efforts have been focusing on exploring new hypotheses that go beyond the classic amyloid one.

In the last few years, we and others demonstrated that the Ventral Tegmental Area (VTA) is one of the first brain regions to be affected in the early phases of the disease [17–25]. The VTA is a deep midbrain nucleus rich in dopaminergic neurons that innervate several brain regions, including the hippocampus, prefrontal cortex, nucleus accumbens (NAc) and amygdala, and participate in tasks like episodic memory, cognition, object recognition, reward and motivation [26–30]. Thus, dopamine (DA) plays a crucial role in the modulation of both cognitive and non-cognitive functions and failure of its release could explain the manifestation of neuropsychiatric symptoms in the prodromal phase of AD [31, 32].

Indeed, as shown by resting-state functional MRI, mild-cognitive-impairment (MCI) patients demonstrate an early and progressive functional disconnection between the VTA and its projecting areas, which worsens in AD [23]. Moreover, the loss of functional connectivity of the VTA with its targets appears more severe in subjects with neuropsychiatric symptoms [17–20, 22–25, 33, 34], and these patients show an accelerated disease progression [35]. Furthermore, a structural MRI study demonstrated a strict correlation between the VTA volume and both the hippocampal size and memory performance, suggesting that changes in the integrity of the VTA might represent an early marker of future neurodegeneration [19]. Interestingly, an in vivo SPECT imaging study for the DA transporter (DAT scan) demonstrated that the dopaminergic projections from the VTA to the mesocorticolimbic areas, namely the ventral striatum (NAc) and hippocampus, were reduced since the MCI phase of AD [22]. All these studies suggest that alteration of mesolimbic connectivity is a very precocious hallmark that could contribute to the disease progression – as previously hypothesized by functional and structural studies [19, 20, 23] – and could provide a valid biomarker for early prognosis [24].

These clinical neuroimaging findings stem from our previous observations in a validated experimental model of AD, the Tg2576 mouse: we showed that VTA dopaminergic neurons undergo a progressive and selective degeneration prior to the deposition of extracellular Aβ plaques in the hippocampus, and much earlier than the observation of hippocampal cell death [21, 36, 37]. DA neuron loss results in reduced dopaminergic (tyrosine hydroxylase positive, TH^+^ and DAT^+^) innervation and lower DA release in the NAc and hippocampus, and coincides with the insurgence of neuronal, memory and reward deficits [21, 38–40], that can be rescued by brief treatment with L-DOPA or selegiline [21, 36]. In line with our data, alterations in the VTA, reduction of DAT in the hippocampus and loss of TH^+^ neurons were also observed in other mouse models of AD (3 × Tg-AD, APPswe/PS11E9 and 5xFAD) [41–44].

Although the specific causes of the increased vulnerability of VTA DA neurons in AD are still unknown, these neurons have particular physiological features that could explain their higher vulnerability [32, 45–48]. Indeed, these cells are spontaneous pacemakers characterized by long, extensively branched axons [49] that need an efficient axonal transport, high-performing mitochondria and an effective autophagy mechanism to remove aggregated proteins or damaged organelles, to efficiently sustain their firing activity. Of note, we have previously demonstrated that VTA DA neurons from Tg2576 mice suffer from autophagy flux alterations and impairments in autophagosome degradation [50]. In line with the importance of efficient autophagic process for DA neuron survival, a pharmacological treatment to improve the autophagic flux in Tg2576 mice prevents the neurodegeneration and ameliorates the AD phenotype [50–52].

Importantly, impairments in autophagy flux could be the basis for accumulation of damaged mitochondria, key organelles that are vital for intracellular calcium (Ca^2+^) buffering [53], and for reactive oxygen species (ROS) production [54]. Several evidence suggest that block of autophagy flux and mitochondrial alterations are responsible for induction of Apoptosis-Inducing Factor (AIF)-mediated apoptosis both in experimental mouse models of AD and Parkinson’s Disease (PD) [55–58] and in post-mortem samples from AD patients [59, 60]. Moreover, mitochondrial dysfunction and cytosolic free Ca^2+^ elevation have been commonly described in the pathogenesis of AD in both animal models and patients [61–66], and are thought to be the key mediators for the degeneration of Substantia Nigra pars compacta (SNpc) DA neurons in PD [67, 68]. Thus, the ability of cells to efficiently buffer free Ca^2+^, with the help of intracellular mediators such as Ca^2+^-binding proteins, can be pivotal for neuronal survival when the mitochondria fail.

In the present work, we focused on the DA neurons projecting from the VTA to the ventral striatum, one of the main regions affected in prodromal AD [22], to investigate the contribution of dysfunctional mitochondria and altered Ca^2+^ buffering to cell death in Tg2576 mice. We saw that degeneration is evident in the VTA since 3 months of age, whereas the neighboring SNpc DA neurons are totally unaffected by the degenerative process. We identified two principal VTA subnuclei (the paranigral, PN and parabrachial pigmented, PBP) that are similarly affected by the degeneration process, resulting in loss of TH^+^ and DAT^+^ innervation in the NAc and severe reduction of DA release. In line with our previous work showing autophagy flux impairment [50], in these VTA subnuclei the DA neurons of Tg2576 mice show accumulation of swollen and damaged mitochondria, as well as translocation of AIF from the mitochondrial inner membrane to the nucleus. These alterations coincide with functional changes in electrophysiological properties. Given the mitochondrial dysfunction, we next analyzed the expression of the Ca^2+^-binding proteins in VTA dopaminergic neurons, in an effort to understand the link between Ca^2+^-binding protein levels and neuronal survival. Interestingly, at the onset of degeneration, the DA neurons in Tg2576 mice are characterized by Ca^2+^-binding protein (Calbindin-D28K or Calretinin, CB or CR, respectively) overexpression, that also results in an important reduction of cytoplasmic Ca^2+^ levels. Importantly, we show that the levels of CB are higher in Tg2576 dopaminergic neurons lacking signs of apoptosis.

Overall, our results depict a scenario in which alterations in mitochondria function/clearance are involved in the early degeneration and in AIF-mediated apoptosis of VTA DA neurons in the Tg2576 AD mice, and unravel a mechanism by which the overexpression of Ca^2+^-binding proteins might be an early attempt of neurons to impede cytoplasmic Ca^2+^ accumulation.

## Methods

### Animals

Heterozygous Tg2576 mice (APPSWE - Model #1349 TACONIC) [69] and WT littermates of either sex were used at 1, 3 and 6 months of age, as described in the text. All experiments were balanced for sex and genotype. All experimental procedures complied with the ARRIVE guidelines and were carried out in accordance with the ethical guidelines of the European Council Directive (2010/63/EU). Experimental approval was obtained from the Italian Ministry of Health (protocol #535/2019PR).

### Constant potential amperometry of evoked DA

Following halothane anesthesia and decapitation, the brain was rapidly removed and coronal brain slices (250–300 μm) containing the dorsal striatum and NAc core/shell were cut with a vibratome (VT1200S, Leica) in chilled bubbled (95% O_2_, 5% CO_2_) solution containing (in mM): NaCl 124, KCl 3, NaH_2_PO_4_ 1.25, NaHCO_3_ 26, MgCl_2_ 1, CaCl_2_ 2, glucose 10 (~ 290 mOsm, pH 7.4). Slices were incubated for 1 h in aCSF at 32 °C and then transferred at room temperature for at least 30 min before recordings. A single brain slice was transferred to a recording chamber and completely submerged in aCSF (3–4 ml min^− 1^; 32 °C).

Amperometric detection of DA was performed as described in [21]. We used carbon fiber electrodes (30 μm diameter, 100 μm length; CF30/100, World Precision Instruments) positioned near a bipolar Ni/Cr stimulating electrode, at a depth of 50–150 μm into the slice. The electrode was allowed to charge for approximately 10 min prior to recording. The voltage (MicroC potentiostat, World Precision Instruments) between the carbon fibre electrode and the Ag/AgCl pellet was set to 0.55 V. For stimulation we used a bipolar stimulator slightly embedded in the brain slice: in the dorsal striatum, we applied a single rectangular electrical pulse with a DS3 Stimulator (Digitimer) every 5 min along a range of stimulation intensities (20–1000 μA, 20–40 μs duration). The stimulation in the NAc core/shell was performed by 5 stimuli (train duration 200 ms, at the same range of intensities as above). In response to an increasing stimulation protocol, a plateau of DA release was reached at the maximal stimulation intensity (1000 μA, 40 μs). Signals were digitized with Digidata 1440A attached to a computer running pClamp 10 (Molecular Devices). Electrode calibration was performed at the end of each experiment by bath-perfused DA (0.3–10 μM) at identical experimental conditions (i.e. temperature, bath flow, pH etc., as the experiments); under these conditions, the sensibility of the carbon electrodes was roughly 100 pA/μM.

### Immunofluorescence

Mice were anaesthetized with Rompun (20 mg ml^− 1^, 0.5 ml kg^− 1^, i.p., Bayer) and Zoletil (100 mg ml^− 1^, 0.5 ml kg^− 1^, Virbac) and perfused transcardially with 50 ml saline followed by 50 ml of 4% paraformaldehyde in Phosphate Buffer (PB; 0.1 M, pH 7.4). The brains were removed and post-fixed in paraformaldehyde at 4 °C and then immersed in 30% sucrose solution at 4 °C until sinking. For immunofluorescence, brains were cut into 30 μm-thick coronal sections using a cryostat and the slices were collected in PB.

For retrograde labelling (see below), sections containing the dorsal striatum and NAc core/shell or the VTA were incubated with Neurotrace-640/660 or Anti-TH antibody, respectively, in PB-Triton 0.3% overnight. After three washes in PB, VTA sections were incubated with secondary antibody.

For TH and DAT fiber analysis, sections containing dorsal and ventral striatum (NAc core and shell) were incubated with the primary antibodies in PB-Triton 0.3% for two nights. After three washes in PB, sections were incubated with secondary antibodies.

For TH/CB and TH/CR immunofluorescent labelling, the selected sections containing the VTA were processed with the primary antibodies in PB-Triton 0.3% for two nights. After three washes in PB, sections were incubated with secondary antibodies. For 3D reconstruction, images were taken as Z-stacks and these Z-stack images were then processed by maximum intensity projection.

For TH/AIF immunofluorescent labelling, after heat-mediated antigen retrieval (10 mM sodium citrate and 0.05% Tween 20, pH 6.0; 10 min of microwaving, as in [70]), sections were incubated with primary antibodies (prepared in 2% BSA in PBS containing 0.1% Triton X-100) for two nights. After three washes in PB, sections were incubated with secondary antibodies in blocking solution for 2 h.

For analysis of biocytin-filled neurons, electrophysiology slices bearing recorded neurons loaded with biocytin (see below) were fixed by immersion in 4% paraformaldehyde in 0.1 M PB for overnight fixation (4 °C). Then, slices were immersed in 30% sucrose solution at 4 °C until sinking. Slices were cut into 80 μm-thick coronal sections using a microtome and the slices were incubated with primary antibody and 555-conjugated Streptavidin in PB-Triton 0.3% and then incubated for 2 h at room temperature with secondary antibody.

For TUNEL assay, brains were cut into 15 μm-thick coronal sections and brain sections were treated according to the manufacturer’s protocol (Click-iT™ Plus TUNEL Assay with Alexa 488 Fluor™ dye; Invitrogen, #C10617), as in [71]. Afterwards, sections were incubated with primary antibodies.

All samples were acquired with the same laser settings. For quantitative analysis, images were collected from at least 3–4 slices processed simultaneously from VTA and exported for analysis with ImageJ software. A range of approximately 10–15 neurons were randomly analyzed per slice.

Primary antibodies: TH (1:1000; ABCAM;112; RRID: AB_297840 or 1:700; Millipore; #MAB318; RRID: AB_2201528); Calbindin-D28K (1:200; Sigma Aldrich; #C9849; RRID: AB_476894); Calretinin (1:150; Millipore; #MAB1568; RRID: AB_94259); AIF (1:200; Millipore; #AB16501; RRID: AB90857); DAT (1:400; Chemicon; #MAB369; RRID: AB_2190413).

Secondary antibodies: Alexa Fluor 488 donkey anti-mouse IgG (1:200; Thermo Fisher Scientific; #R37114; RRID: AB_2556542); Alexa Fluor 555 donkey anti-rabbit IgG (1:200; Thermo Fisher Scientific; # A31572; RRID: AB_162543); Alexa Fluor 647 donkey anti-mouse IgG (1:200; Thermo Fisher Scientific; #A31571; RRID: AB_162542); Alexa Fluor 488 donkey anti-rat IgG (1:200; Thermo Fisher Scientific; # A21208; RRID: AB_2535794), Streptavidin, Alexa Fluor 555 Conjugate (1:750; Invitrogen #S32355; RRID: AB_2571525); Neurotrace-640/660 (1:200; Thermo Fisher Scientific; # N21483; RRID: AB_2572212).

Sections for immunofluorescence were counterstained with DAPI, coverslipped with Aqueous Mounting Media (Sigma-Aldrich) and examined under a confocal laser-scanning microscope (Nikon Eclipse Ti2-A). The specificity of the immunofluorescence labelling was confirmed by the omission of primary antibodies and the use of normal serum instead (negative controls).

### Retrograde labeling

For retrograde tracing of striatal/NAc-projecting neurons, WT mice were anaesthetized with Rompun (20 mg ml^− 1^, 0.5 ml kg^− 1^; Bayer) and Zoletil (100 mg ml^− 1^, 0.5 ml kg^− 1^; Virbac; i.p.) and positioned in a stereotaxic apparatus, before a burr hole was made to the skull under aseptic conditions. Red retrobeads (Lumafluor, Naples, USA) were injected bilaterally under stereotaxic control in the NAc medial shell (bregma: 1.8 mm, ML: ± 0.4 mm, DV: 4.1 mm; 120 nl beads), NAc core (bregma: 1.5 mm, ML: ± 0.9 mm, DV: 4.1 mm; 120 nl beads) and dorsal striatum (bregma: 1 mm, ML: ± 1.6 mm, DV: 2.3 mm; 120 nl beads) [72]. The retrobeads were infused using a 1 μl Hamilton syringe (Neuros7001; Hamilton; #65458–01) by slow pressure lasting 10 min using a Pump 11 Elite Nanomite Syringe Pump (Harvard Apparatus; flux 12 nl/min) to allow for diffusion into the target brain area. Following infusion, the needle remained in place for at least an additional 8 min to prevent backflow, before being slowly retracted. After surgery the skin was sutured, and mice were returned to their home cage and monitored during recovery. Each animal was injected at postnatal day 60 (P60) and analyzed with immunofluorescence 1 month after surgery (~P90). Data from animals showing a misplaced injection site were discarded.

### Stereological analysis

Sections processed for immunofluorescence were used for obtaining estimates of numbers of TH^+^ neurons in the midbrain (SNpc and VTA; see [50, 73]) or numbers of TH^+^ neurons containing calbindin (TH^+^CB^+^) or calretinin (TH^+^CR^+^) in the VTA. The boundaries of the areas used for counting were defined by TH staining, and area distinction was performed according to published guidelines [72] and as described in the text. We applied an optical fractionator stereological design (bilateral count) using the Stereo Investigator System (MicroBrightField Europe e.K.). A stack of MAC 5000 controller modules (Ludl Electronic Products, Ltd) was interfaced with an Olympus BX50 microscope with a motorized stage and a HV-C20 Hitachi digital camera with a Pentium II PC workstation. A three-dimensional optical fractionator counting probe (x, y, z dimension of 50 × 50 × 25 μm) was applied. The brain area of interest was outlined using the 5x objective and neuronal cells were marked with a 100x oil-immersion objective. Neurons were considered TH^+^, CB^+^ or CR^+^ if they showed cytoplasmatic immunoreactivity.

The total neuron numbers were estimated according to the formula (eq. 1):

1 $$N= SQ\times \frac{1}{ssf}\times \frac{1}{asf}\times \frac{1}{tsf}$$

where SQ represents the number of neurons counted in all optically sampled fields of the area of interest, ssf is the section sampling fraction, asf is the area sampling fraction and tsf is the thickness sampling fraction.

### Transmission Electron microscopy (TEM)

Analysis was performed in fields employed from a previous study [50]. Details of tissue preparation, post-embedding procedure, ultrathin cutting, and TH staining are fully reported in [50]. Briefly, following perfusion, brains were cut on a vibratome in 50 μm horizontal sections containing the midbrain and collected in PB until processing [74]. Sections were obtained through the dorso-ventral extent of the VTA, resulting in 12–13 consecutive sections and processed with an osmium-free embedding method [74]. VTA chips were sectioned with an ultramicrotome (60–80 nm), mounted on 300 mesh nickel grids and processed for immunogold labeling [74, 75], with primary antibody anti-TH (1:200; Abcam; #AB1542; RRID: AB_90755), and secondary antibody conjugated to 18 nm gold particles (1:20; Jackson, 713–215-147; RRID: AB_2340734).

Ultrathin sections were examined with a Philips CM10 electron microscope coupled to a MegaView-II high resolution CCD camera (Soft Imaging System). Identification of ultrastructural profiles was based on established morphological criteria [76].

Mitochondria morphology was analyzed in the same TH^+^ neurons recognized and studied in [50], and identification of normal and swollen/vacuolated mitochondria was performed according to established morphological criteria [77–79]. In more detail: normal-appearing rounded and oval-elongated-shaped mitochondria were characterized by a smooth outer membrane and an inner folded membrane that formed the cristae, which was filled with dense matrix. In normal mitochondria, the cristae were well preserved, and the electron density of mitochondrial matrix appeared as regular (representative examples of normal mitochondria (n) are illustrated in the upper, middle, and lower panels from WT and Tg mice). Swollen and vacuolated-appearing mitochondria exhibited a dilatation of the intermembrane space with a splitting of the outer and inner membranes including micro­vacuolization of the inner compartment and an irregular appearance of matrix and cristae (examples of swollen mitochondria (s) are illustrated in the upper, middle, and lower panels from WT and Tg mice). Vacuolated mitochondria included vacuoles deriving from the dilatation of the intermembrane space localized between the outer and inner membranes, vacuoles in the matrix or cristae (see (v) in examples illustrated in Fig. 3), and finally large vacuoles (referring to (V) in representative panels) containing granular or amorphous substance or characterized by empty spaces of various sizes.

### Electrophysiological recordings

Solutions for brain slicing were prepared fresh as in [80], and kept at 4 °C for 5 days at most. All were saturated with a mixture of 95% O_2_, 5% CO_2_ prior to usage; pH was checked daily to be between 7.3 and 7.4. Acute coronal brain slices containing the midbrain were obtained following halothane anesthesia and transcardiac perfusion with a solution containing (in mM): 92 NMDG, 2.5 KCl, 1.2 NaH_2_PO_4_, 30 NaHCO_3_, 20 HEPES, 25 Glucose, 5 Na-Ascorbate, 2 Thiourea, 3 Na-Pyruvate, 10 MgSO_4_, 0.5 CaCl_2_. The brain was rapidly removed, and slices (240 μm) were cut with a vibratome (VT1200S, Leica) in chilled (4 °C) perfusion solution. After cutting, brain slices containing the midbrain were transferred to a holding chamber with the same NMDG-based solution, and left to recover at 34 °C for 25 min during which we gradually increased sodium concentration [80].

After recovery, slices were transferred to a long-term holding chamber containing (in mM): 92 NaCl, 2.5 KCl, 1.2 NaH_2_PO_4_, 30 NaHCO_3_, 20 HEPES, 25 Glucose, 5 Na-Ascorbate, 2 Thiourea, 3 Na-Pyruvate, 2 MgSO_4_, 2 CaCl_2_, where they were kept for the entire experimental session at room temperature.

A single slice was placed into a recording chamber of an upright microscope (Axioskop 2-FS; Zeiss) and continuously perfused (2.5 ml sec^− 1^, 32 °C) with a solution containing (in mM): 124 NaCl, 2.5 KCl, 1.2 NaH_2_PO_4_, 24 NaHCO_3_, 5 HEPES, 12.5 Glucose, 2 MgSO_4_, 2 CaCl_2_. The midbrain was identified at 4x magnification and single neurons were visualized using a magnification of 60x.

Whole-cell patch-clamp recordings were performed in the PN. The target cell was approached with a glass capillary (TW-150F-4, World Precision Instruments) with a resistance of 3.5–4.5 MΩ and filled with a filtered solution containing (in mM): 120 K-Glu, 20 KCl, 0.2 EGTA, 10 HEPES, 2 MgCl_2_, 4 Mg-ATP, 0.3 Na-GTP, 10 Phosphocreatine, 0.2% Biocytin, pH 7.30. After the formation of a giga-seal and membrane rupture, the cell’s Cm was taken online from the membrane seal test function of pClamp 10.3 (− 5 mV step, 15 ms).

To analyze the rheobase current (the minimum injected current able to generate the first AP) and AP shape, cells were kept in current clamp mode at − 60 mV by direct current injection and short (200 ms) depolarizing steps (Δ = 5 pA) were injected until the cell fired [81]. The rise time and the decay time of the first AP were calculated as the x-axis distance between the threshold potential and the peak potential, and between the peak potential and the minimum value reached during the hyperpolarization phase, respectively. The AP amplitude was calculated as the y-axis distance between threshold and peak. The width was defined as the duration between the threshold and the equipotential point found on the repolarization phase of the AP. The AHP was the most negative membrane potential during the AP. The threshold was determined from phase-plane plots of dVm/dt *vs* Vm (‘phase plots’), corresponding to the membrane potential value at which dVm/dt increased suddenly and developed with a monotonic rise [21, 37].

A longer current-clamp protocol was applied (600 ms) with steps of 50 pA, starting from − 200 pA up to + 400 pA, to evaluate the I/V relationship and sag at sub-threshold potentials, and the number of APs at above-threshold potentials. Membrane resistance (R_m_) was calculated from the slope of the linear regression of I/V curves. The sag was the difference between the peak and the steady-state potential taken from the − 200 pA step.

Voltage-clamp was used to study different DA neuron conductances (see Table 1): a 1 s protocol with 4 hyperpolarizing steps from − 60 to − 120 mV (Δ = 20 mV) was applied to study the amplitude of the I_h_ current. The I_h_ was calculated as the difference between the peak current value and the steady-state current reached during the − 120 step. For the SK current we applied a voltage-clamp protocol consisting of a 100 ms depolarization from − 60 to 0 mV, and SK current was expressed as a peak right after the depolarizing current. Peak currents are reported in Table 1; similar results between WT and Tg2576 neurons were obtained for current densities (data not shown).

**Table 1:** Electrophysiological properties of VTA DA neurons in the PN nucleus of 3-month-old mice

Parameter	WT	Tg2576	Statistics^#^
**Membrane Capacitance (pF)**	45.95 ± 4.09 (22)	30.39 ± 2.10 (20)	*p* = 0.0020
**Membrane Resistance (MΩ)**	344.10 ± 36.64 (16)	407.8.10 ± 39.07 (14)	*p* = 0.2445
**AP Amplitude (mV)**	48.99 ± 6.04 (10)	49.54 ± 3.59 (11)	*p* = 0.9384
**AP Rise Time (ms)**	0.81 ± 0.11 (10)	1.06 ± 0.15 (11)	*p* = 0.2018
**AP Decay Time (ms)**	3.77 ± 0.31 (10)	5.45 ± 0.94 (11)	*p* = 0.1170
**AP Width (ms)**	2.01 ± 0.20 (10)	2.78 ± 0.54 (11)	*p* = 0.2040
**Ih Current (pA)**	−3.007 ± 1.382 (13)	−9.487 ± 3.979 (12)	*p* = 0.1564
**SK Current (pA)**	85.82 ± 21.08 (15)	65.35 ± 16.21 (12)	*p* = 0.4488

Passive membrane properties, AP parameters and amplitude of I_h_ and SK currents (mean ± s.e.m) of VTA DA neurons located in the PN, from 3-month-old WT and Tg2465 mice. The number of analyzed neurons is shown in brackets. ^#^ Welch’s *t*-test (for WT *vs* Tg2576)

During all experiments, the membrane access resistance was repeatedly monitored and recordings in which it exceeded over 25% were discarded. No liquid junction potential correction was applied.

Single-unit extracellular recordings were performed under a 4x objective. The electrode was filled with extracellular solution and moved in the VTA (PN and PBP) until firing was detected, to allow for non-invasive recording of spontaneous firing. Firing from putative DA neurons was identified based on the well-known characteristics of slow and regular firing for these cells [45]. Spikes were recorded on *I* = 0 mode, with high-pass (0.5 Hz) and low-pass filtering (1 kHz). DC components were also highpass-filtered by enabling 300 Hz AC coupling [45, 82].

All signals were amplified with an Axon 700B amplifier, digitized at 20 kHz with a Digidata 1400A and computer-saved using Clampex 10.3 (all from Molecular Devices, Sunnyvale, CA).

### Total protein extraction and Western-blot analysis

The midbrain was dissected from the entire brain and stored at − 80 °C until the day of the experiment. Tissue was homogenized in RIPA buffer containing (in mM) 50 Tris-HCl pH 7.5, 150 NaCl, 5 MgCl_2_, 1 EDTA, 1% Triton X-100, 0.25% sodium deoxycholate, 0.1% SDS, 1 sodium orthovanadate, 5 b-glycerophosphate, 5 NaF and protease inhibitor cocktail, and incubated on ice for 30 min [50, 83]. The samples were centrifuged at 15,000 g for 20 min and the protein concentration of the supernatant was determined by the Bradford method.

Proteins were applied to SDS–PAGE and electroblotted on a polyvinylidene difluoride membrane. Blotting analysis was performed using a chemiluminescence detection kit. The relative levels of immunoreactivity were determined by densitometry using ImageJ.

Primary antibodies: Calbindin-D28K (1:200, Sigma Aldrich; #C9849; RRID: AB_476894); Calretinin (1:200, Millipore; #MAB1568; RRID: AB_94259); Actin (1:10000; Sigma Aldrich; #A5060; RRID: AB_476738).

Secondary antibodies: goat anti-mouse IgG (1:3000; Bio-Rad; #1706516; RRID: AB_11125547), goat anti-rabbit IgG (1:3000; Bio-Rad; #1706515; RRID: AB_2617112).

Membranes were stripped using Re-Blot Plus Strong Solution (Millipore) for 15 min at room temperature.

For each age, both genotypes were analyzed simultaneously. The different ages were analyzed in different blots.

### Ca^2+^ microfluorometry

Measurements of intracellular free Ca^2+^ were performed as previously described [73] during whole-cell recordings using pipettes (2–5 MΩ) filled with (mM): 145 K-gluconate, 0.1 CaCl_2_, 2 MgCl_2_, 10 HEPES, 0.75 mM EGTA, 0.25 Fura-2 K^+^ salt (ab142777; Abcam), 2 ATP-Mg^2+^ and 0.3 GTP-Na^+^ (pH 7.3, 280 mOsm). Cells were illuminated using METAFLUOR (Molecular Devices), which provided 340 and 380 nm excitation wave-lengths. Fluorescence ratios (R) were taken directly from the software based on the specific fluorescence values F_340_ and F_380_ emitted by the region of interest (ROI) and background (BK) at 340 and 380 nm excitation wavelengths, using the following eq. 2:

2 $$R=\frac{F_{340}\times ROI-{F}_{340}\times BK}{F_{380}\times ROI-{F}_{380}\times BK}$$

### Sample size, randomization and blinding

The sample number for each group and for each experiment was determined according to our earlier studies. In more detail, power analysis was performed by G*Power software (version 3.1.9.7) using as input values a power of 0.8, errors of 0.05 and standard deviations of WT and Tg2576 obtained from our previous publications where similar experiments were performed using this mouse model.

Randomization (i.e. how the different mice born from the same litter were ‘destined’ for the different experimental groups) was performed using a random number table, by matching for sex and age.

Data were collected by researchers blinded to the genotype of each animal; un-blinding occurred after data analysis.

The experimental units used for each experiment are described in more detail in the Figure legends. Briefly, these were: the total number of DA recordings for amperometry, the number of recorded neurons for electrophysiology, the number of mice for Western blot, stereology and confocal microscopy, and the number of neurons for TEM and TUNEL assay.

### Statistical analysis

Statistical analysis was performed with GraphPad Prism (v7.00).

In all cases where 2 independent factors were examined (i.e age and genotype), data were analyzed by Two-Way ANOVA, followed by with Tukey’s or Sidak’s post-hoc tests in cases of interaction across parameters. In cases when no interaction was observed between the independent factors, we used simple *t*-tests: data were first checked for normality by using the D’Agostino and Pearson or Shapiro-Wilk normality tests and analyzed accordingly with two-tailed parametric (Unpaired *t*-test, Welch’s *t-*test) or non-parametric tests (Mann–Whitney test). See figure legends for more details. Values of *p* ≤ 0.05 were considered to be statistically significant.

In the box-and-whisker plots, the center line denotes the median value, edges are upper and lower quartiles, whiskers show minimum and maximum values and points are individual experiments.

All other data are presented as mean ± s.e.m.

## Results

### The mesolimbic core and medial shell DA neurons degenerate early in Tg2576 mice

To study the cellular mechanisms related to cell death in dopaminergic neurons of Tg2576 mice, in the present study we first had to identify subregions in the midbrain that are particularly susceptible to degeneration. To monitor DA release, we recorded evoked DA by electrical stimulation of the mesolimbic or mesostriatal dopaminergic pathways: we focused the amperometric recordings in the NAc core and medial shell – these being the principal mesolimbic targets of VTA DA neurons, precociously affected in MCI patients [22] – and in the dorsal striatum, the main output of neighboring SNpc neurons [46]. We then combined this information with retrograde tracing from the target areas, and with stereological counting of midbrain DA neurons, identified by TH staining.

**Fig. 1:**
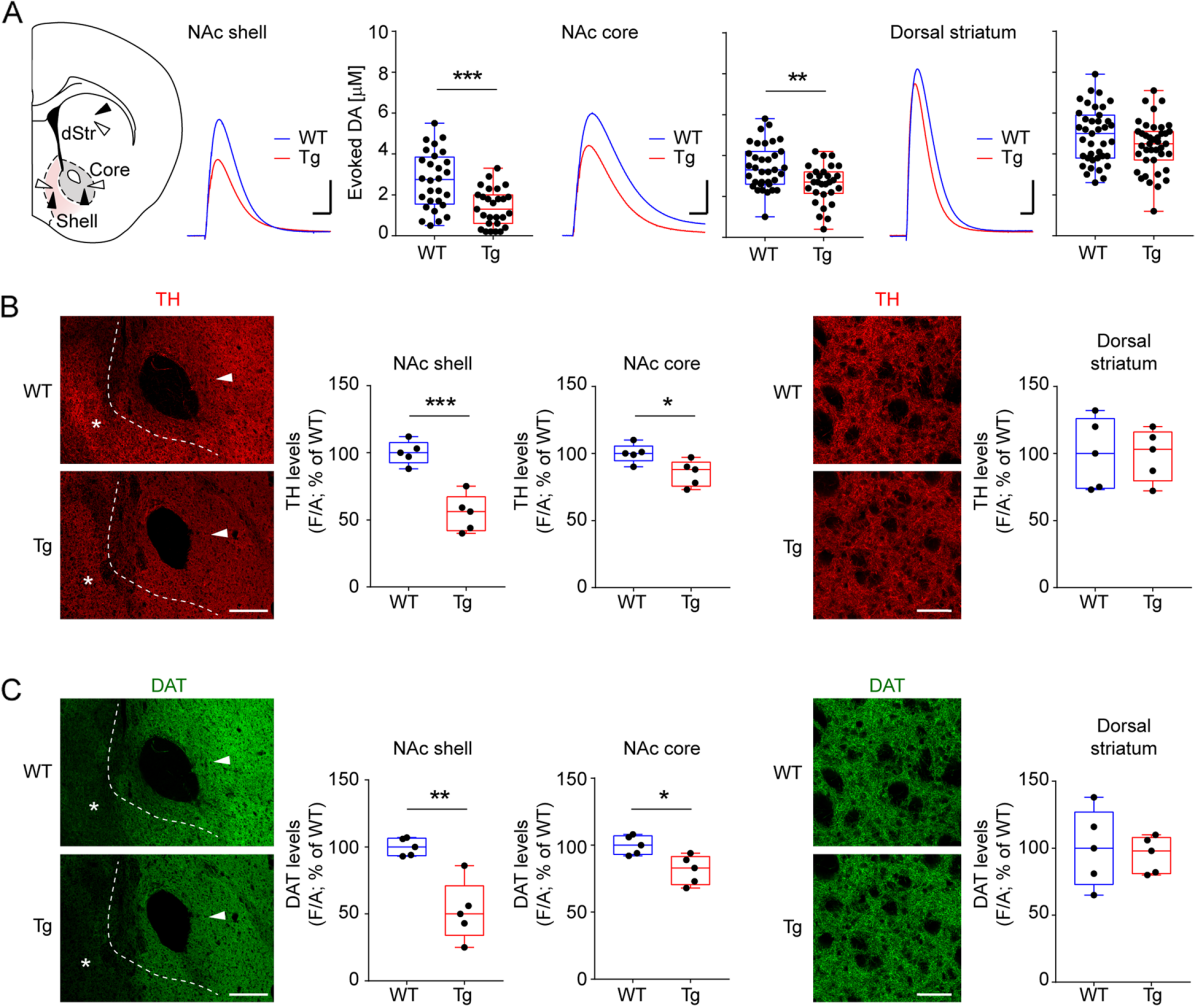
Reduced dopamine and dopaminergic innervation in Tg2576 NAc core and shell since 4 months of age. **A** Example, representative traces (scale: 100 pA; 200 ms) and evoked DA concentration in NAc shell, NAc core and striatum (WT: *n* = 28–39 observations from 10 to 13 slices, 4 mice; Tg2576: *n* = 27–39 observations from 10 to 13 slices, 4 mice) in 4-month-old WT and Tg2576 mice recorded with a carbon fiber electrode of equal calibration (NAc shell; Unpaired *t*- test with Welch’s correction, ***p = 0.0001; NAc core: Unpaired *t*-test, **p = 0.0015). The schematic on the left shows the placement of the stimulating (black arrowheads) and carbon fiber electrodes (white arrowheads) used for the recordings in the three brain regions. **B** Representative immunofluorescent labelling (scale bar, 200 μm) for TH in 4-month-old WT and Tg2576 mice and graphs showing densitometric values of TH levels. *Left panels*: NAc coronal sections showing the NAc shell (asterisk; *n* = 5 per genotype. Unpaired *t*-test: ***p = 0.0003) and NAc core (arrowhead; *n* = 5 per genotype. Unpaired *t*-test: *p = 0.0245). *Right panels*: dorsal striatum (scale bar, 50 μm. *n* = 5 per genotype). **C** As in B, but showing immunofluorescent labelling (scale bar, 200 μm) for DAT in NAc shell (asterisk; *n* = 5 per genotype. Unpaired *t*-test: **p = 0.0017), NAc core (arrowhead; *n* = 5 per genotype. Unpaired *t*-test: *p = 0.0123) and dorsal striatum (scale bar, 50 μm. *n* = 5 per genotype). In this and all other Figures, in box-and-whisker plots the center line shows the median value, edges are upper and lower quartiles, whiskers show minimum and maximum values, and each point is an individual experiment

By amperometric recordings we observed a reduction of evoked DA in 4-month-old Tg2576 mice in the NAc medial shell and core compared to WT littermates, whereas the dorsal striatum was only slightly, yet insignificantly, affected (Fig. 1A). Of note, at 3 months of age DA levels in all these areas are still comparable to controls (Fig. S1A). To try to explain the discrepancy in the amperometric data between 3- and 4-months of age, we examined the dopaminergic innervation in the NAc core, shell and dorsal striatum by analyzing the levels of TH and DAT at these ages. Indeed, at 3 months of age we observed no difference for TH or DAT staining at any of the analyzed regions between WT and Tg2576 mice (Fig. S1B,C); on the other hand, at 4-months of age there was a significant reduction in fluorescence intensity in the NAc core and shell of Tg2576 mice, but no differences were observed in the dorsal striatum compared to control animals (Fig. 1B,C). Overall, these data show that at 3-months of age there appear to be no appreciable differences in dopaminergic innervation or DA release in the ventral striatum between WT and Tg2576 mice, but these become more apparent with age progression.

**Fig. 2:**
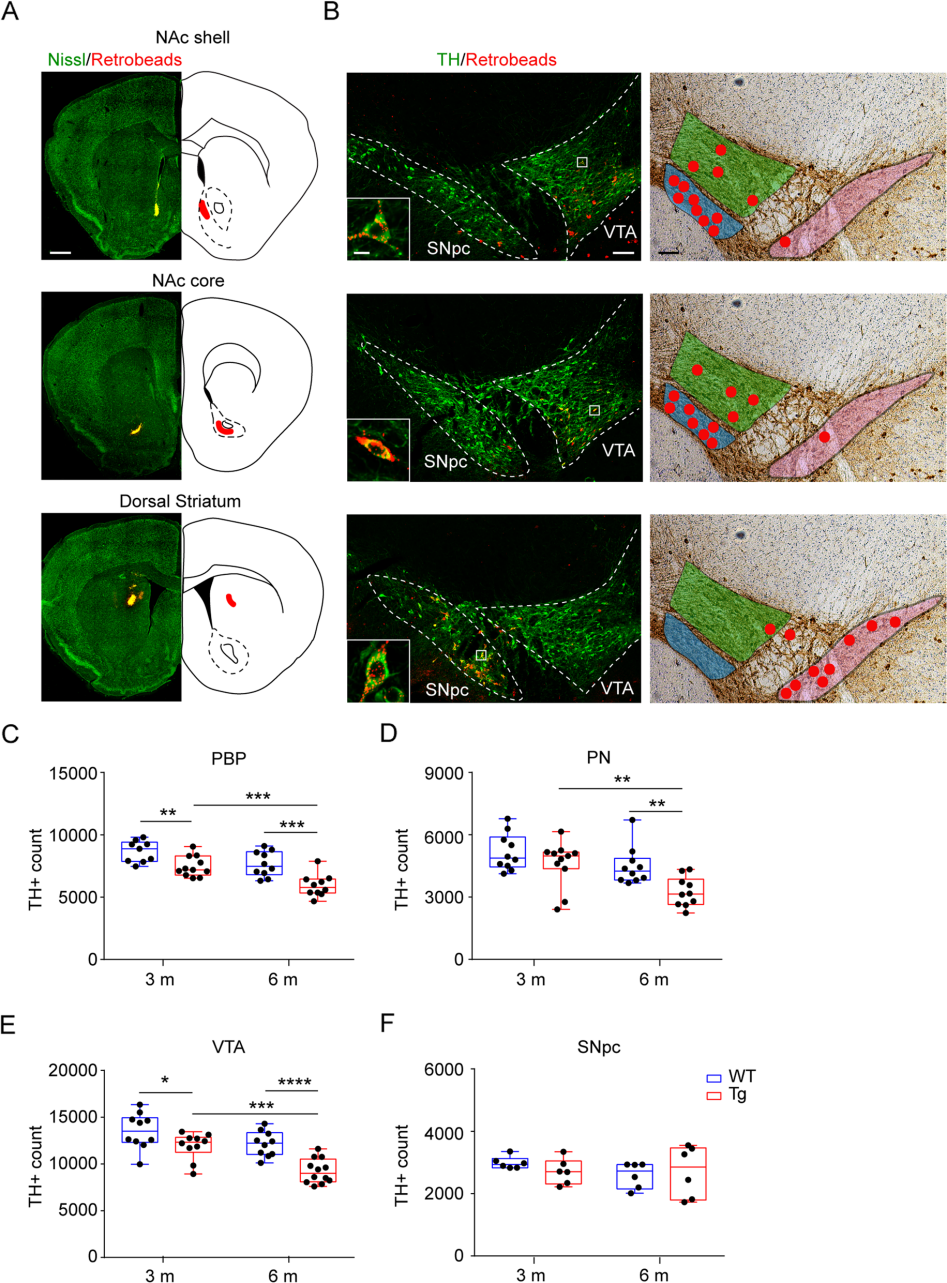
Selective degeneration of Tg2576 DA neurons projecting to NAc core and shell since 3 months of age. **A** Retrograde tracing of midbrain neurons projecting to ventral and dorsal striatum 3 weeks from the injection. *Left panels*: injection sites of red fluorescent beads (Retrobeads) in coronal sections of NAc shell, NAc core and dorsal striatum (green-Nissl counterstained; scale: 500 μm). *Right panels*: Schematic representation of the corresponding midbrain regions, with injected areas marked. **B** Anatomical position of retrogradely labeled TH^+^ neurons in the midbrain. *Left panels:* confocal images of retrogradely labeled DA neurons from coronal midbrain sections containing VTA and SNpc (red + green; scale: 100 μm), and inset showing TH^+^ cells (green) in the VTA PBP (upper panel), VTA PN (middle panel), and SNpc (bottom panel) labeled with red beads from NAc shell, core and dorsal striatum, respectively (scale: 2 μm). *Right panels*: schematic images of coronal midbrain sections containing VTA and SNpc (scale: 100 μm). The two VTA subnuclei and SNpc are differently colored (paranigral (PN): blue; parabrachial (PBP): green; SNpc: pink). Red dots represent the position of individual retrobeads-TH^+^ neurons in VTA and SNpc accumulated from three different experiments. **C-D** Stereological quantification of TH^+^ cell numbers in (**C**) PBP and (**D**) PN from 3- and 6-month-old WT and Tg2576 mice. **C** PBP (*3 months*: WT: *n* = 9; Tg2576: *n* = 11; *6 months*: WT: *n* = 10; Tg2576: *n* = 10 mice. Two-way ANOVA for Genotype vs Age: Interaction: F_1,36_ = 0.81, *p* = 0.3760; Genotype: F_1,36_ = 26,64, *p* < 0.0001; Age: F_1,36_ = 19.85, *p* < 0.0001. WT 3 m vs Tg2576 3 m: Unpaired *t*-test **p = 0.0047; WT 6 m vs Tg2576 6 m: Unpaired *t*-test ***p = 0.0007; Tg2576 3 m vs Tg2576 6 m: Unpaired *t*-test ***p = 0.0007). **D** PN (*3 months*: WT: *n* = 10; Tg2576: *n* = 11; *6 months*: WT: *n* = 10; Tg2576: *n* = 10 mice. Two-way ANOVA for Genotype vs Age: Interaction: F_1,37_ = 1.437, *p* = 0.2382; Genotype: F_1,37_ = 9.63, *p* = 0.0037; Age: F_1,37_ = 12.53, *p* = 0.0011. WT 6 m vs Tg2576 6 m: Mann-Whitney test **p = 0.0021; Tg2576 3 m vs Tg2576 6 m: Mann-Whitney test **p = 0.0048). **E-F** Stereological quantification of TH^+^ cell numbers in (**E**) VTA and (**F**) SNpc from 3- and 6-month-old WT and Tg2576 mice. **E** VTA (*3 months*: WT: *n* = 10; Tg2576: *n* = 10; *6 months*: WT: *n* = 10; Tg2576: *n* = 12 mice. Two-way ANOVA for Genotype vs Age: Interaction: F_1,38_ = 1.868, *p* = 0.1797. WT 3 m vs Tg2576 3 m: Unpaired *t*-test: *p = 0.0466; WT 6 m vs Tg2576 6 m: Unpaired *t*-test: ****p < 0.0001; Tg2576 3 m vs Tg2576 6 m: Unpaired *t*-test: ***p = 0.0002). **F** SNpc (*3 months*: WT: *n* = 6; Tg2576: *n* = 6; *6 months*: WT: *n* = 6; Tg2576: *n* = 6 mice)

We hypothesized that the amperometric and imaging results, showing location-specific loss of DA and dopaminergic innervation, would mirror the degree of DA neuron degeneration in the midbrain, likely being stronger for VTA neurons projecting to the NAc medial shell and core. Thus, we next mapped the neuroanatomical distribution of retrogradely-labelled DA neurons from different limbic regions: NAc medial shell, NAc core and dorsal striatum (Fig. 2A). As previously demonstrated by other groups [46, 84–86], we observed that VTA DA neurons that project to the NAc medial shell and NAc core are mainly localized in the PN and PBP nuclei of the VTA (Fig. 2B); instead, the dorsal striatum is almost exclusively innervated by the dopaminergic neurons localized in the SNpc. Of note, we observed a wide overlap between the localization of mesolimbic NAc core- and NAc medial shell-projecting TH^+^ neurons, that are intermingled in the PN and PBP in WT animals.

In line with the amperometric data, a quantitative analysis of TH^+^ neuron numbers confirmed that both VTA subnuclei were affected by progressive neurodegeneration (Fig. 2C,D). Indeed, by comparing WT and Tg2576 neurons we overall confirmed a strong reduction in the numbers of VTA neurons of Tg2576 mice, that was evident since 3 months of age and became even stronger at 6 months of age. Moreover, within the Tg2576 genotype, TH^+^ neuron numbers continued to decrease, indicating an age-dependent neurodegeneration (Fig. 2E). On the other hand, TH^+^ neurons in the neighboring SNpc did not show any degeneration in Tg2576 mice at both the ages examined (Fig. 2F). This confirms our earlier observations from Tg2576 mice that the degenerative process is selective for VTA neurons, whereas SNpc TH^+^ neurons are intact, at least until 6 months of age, with no evidence of either apoptosis or neuroinflammation, unlike the VTA [21]. Taken together, our current data demonstrate that mesolimbic DA neurons from the VTA, located in the PN and PBP, show progressive neuronal degeneration in Tg2576 mice, that is translated into significant loss of evoked DA in the NAc medial shell and core.

### DA neurons in the VTA of Tg2576 mice show reduced mitochondrial integrity

**Fig. 3:**
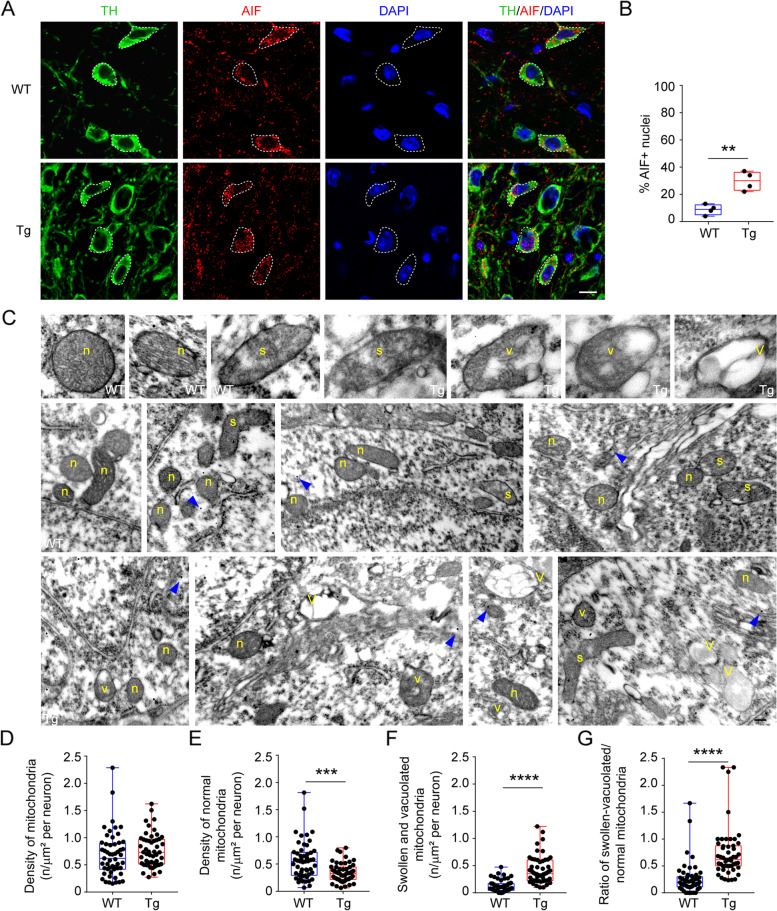
VTA DA neurons in the Tg2576 mice show mitochondrial alterations at 3 months of age. **A** Representative confocal images of AIF labeling in TH^+^ neurons from WT and Tg2576 mice at 3 months of age (scale: 10 μm). **B** The graph shows the % of AIF^+^ nuclei in DA neurons of the VTA (*n* = 4 mice per genotype; Unpaired *t*-test: **p = 0.0018). **C** TEM representative images of normal, swollen, and vacuolated mitochondria (upper row; original magnification 92.000x) from osmium-free TH-stained post-embedded VTA of 3-month-old WT (panels of middle row) and Tg2576 mice (lower row; original magnification 24.000x). Normal-appearing mitochondria (n in all panels) display a smooth outer membrane and well-preserved cristae filled with a regular electron density of the mitochondrial matrix. Swollen (s) and vacuolated-appearing (v) mitochondria exhibit dilation of the intermembrane space, vacuolization of the inner compartment, and an irregular appearance of matrix and cristae. Entirely vacuolized mitochondria (V) contain an amorphous substance or appear as empty vacuoles of various sizes. Although mitochondrial swelling and vacuolization are detectable in WT neurons, Tg2576 neurons show an increase of mitochondria at different stages of swelling and/or vacuolization (plots** D-G**). In all TEM panels, blue arrowheads point to TH-coding gold particles (scale bar: 30 nm for upper panels, and 130 nm for middle and lower panels; WT: *n* = 53 TH^+^ neurons; from 3 mice; Tg2576 mice: *n* = 51 TH^+^ neurons, from 3 mice. **D** Total density of mitochondria (0.68/μm^2^ ± 0.05 and 0.76/μm^2^ ± 0.04); **E** density of normal mitochondria (0.55/μm^2^ ± 0.04 and 0.34/μm^2^ ± 0.02; Mann-Whitney test: ***p = 0.0003), **F** density of swollen/vacuolated mitochondria (0.12/μm^2^ ± 0.01 and 0.42/μm^2^ ± 0.03; Mann-Whitney test: ****p < 0.0001), and **G** ratio of swollen-vacuolated/normal mitochondria (Mann-Whitney test: ****p < 0.0001)

We have previously observed that VTA DA neurons of 3-month-old Tg2576 mice, at the onset of degeneration, suffer from autophagy flux alterations and accumulation of autophagosomes [50]. These deficits could be related to accumulation of damaged organelles, like mitochondria, or of unfolded/aggregated protein, like Aβ. Dysfunctional mitochondria and alterations in mitophagy – the specific autophagy mechanism responsible for removal of these organelles – can be detrimental for neuronal survival by inducing apoptotic processes [87]. Interestingly, a block in autophagy clearance can induce apoptosis via AIF [88], a small protein normally confined to mitochondria that causes cell death by translocating to the nucleus [89]. For this reason, and to better understand the basis of cell death in the VTA, we analyzed AIF expression and cellular localization in Tg2576 DA neurons. In line with the degenerative process occurring in the VTA (see Fig. 2), in 3-month-old Tg2576 mice we found an increased number of TH^+^ cells exhibiting nuclear AIF (Fig. 3A-B).

Of note, AIF release to the cytoplasm is associated with mitochondrial alterations [89, 90]. Thus, to assess mitochondria integrity, in terms of morphology and cristae shape, we used TEM on VTA dopaminergic neurons from 3-month-old mice. In the VTA of post-embedded samples from WT and Tg2576 mice, DA neurons were identified based on the density of cytoplasmic TH-coding particles [50]. Ultrastructural analysis revealed that mitochondria in Tg2576 neurons display different stages of swelling and/or vacuolization, whilst WT mitochondria mostly have well-preserved cristae with a regular matrix (Fig. 3C). Interestingly, although the total number of mitochondria was comparable between groups (Fig. 3D), the density of normal mitochondria was significantly higher in WT than in Tg2576 neurons (Fig. 3E); conversely, the density of swollen/vacuolated mitochondria was higher in Tg2576 mice (Fig. 3F). These data indicate that the ratio of swollen-vacuolated to normal mitochondria was significantly higher in Tg2576 neurons (Fig. 3G), in line with our hypothesis that deficits in autophagic processes in Tg2576 DA neurons might result in the accumulation of damaged mitochondria, contributing to neuronal cell death by AIF-mediated apoptosis.

### Increased neuronal excitability of PN-located DA neurons in Tg2576 mice

**Fig. 4:**
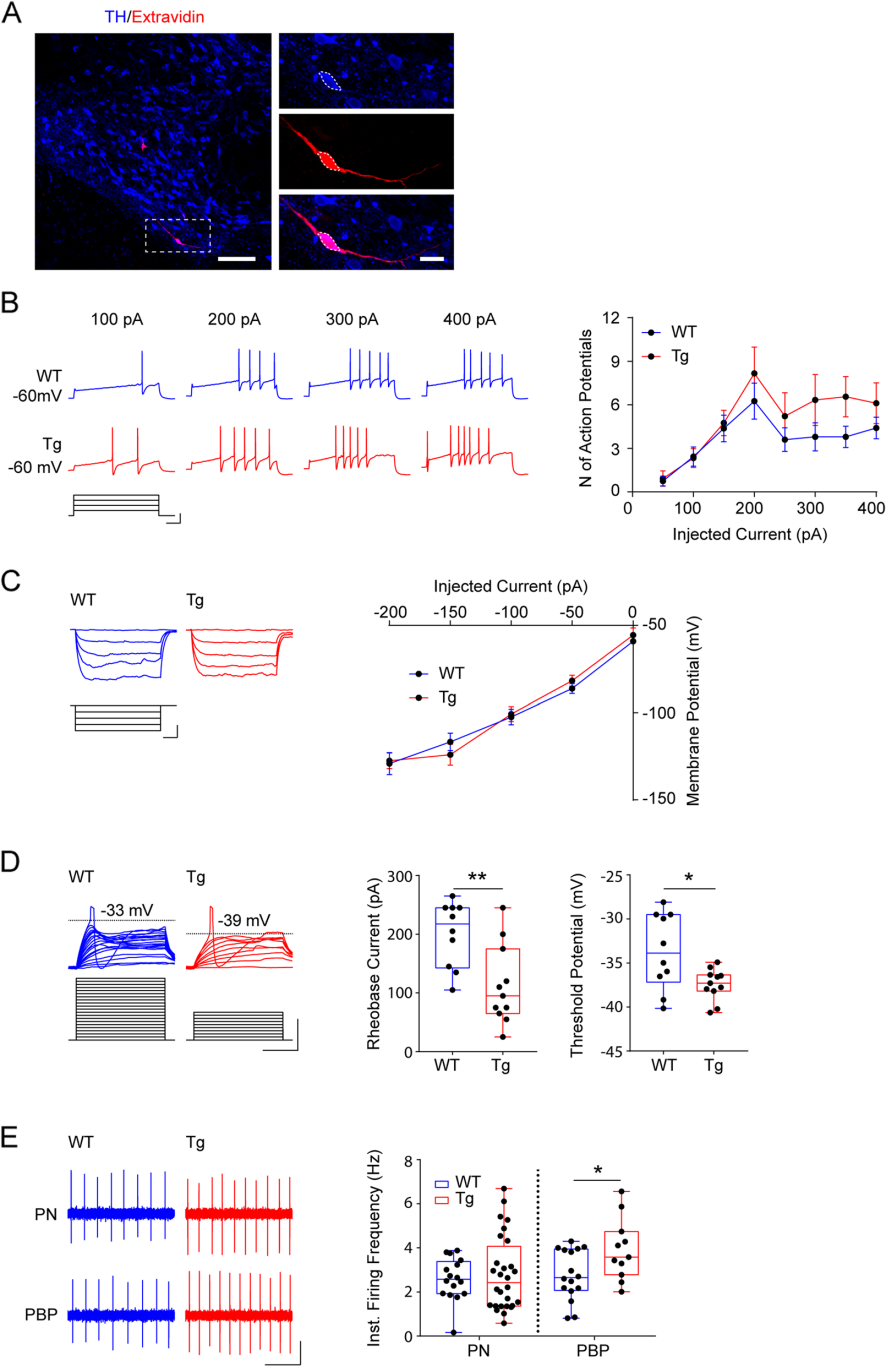
DA neurons in the PN of Tg2576 mice show hyperexcitability at 3 months of age. **A** Representative double immunofluorescent labelling for TH (blue) and biocytin-filled neuron (extravidin; red) in VTA coronal sections (scale: 100 μm). On the right are high magnification images of labelling for TH (blue) and biocytin-filled (red) recorded neurons (scale: 20 μm). **B** Representative AP traces, recorded in current-clamp mode following 50 pA-stepped depolarizing current injections (scale: 100 ms; 20 mV, 200 pA) in PN DA neurons from WT and Tg2576 mice initially held at − 60 mV, and plot demonstrating the average number of AP (± s.e.m.) elicited every 100 pA depolarizing steps (WT *n* = 16 cells; Tg2576 *n* = 12 cells from 10 mice each; two-way ANOVA for genotype vs injected current: interaction: F_7,152_ = 0.4341, *p* = 0.8796; genotype: F_1,152_ = 4.82, *p* = 0.0297; injected current: F_7,152_ = 7.197, *p* < 0.0001). **C** Sub-threshold responses to 50 pA-stepped hyperpolarizations of PN neurons (scale: 100 ms; 20 mV, 100 pA) and mean current/voltage relationship (± s.e.m.; WT: *n* = 16 neurons, 7 mice; Tg2576: *n* = 15 neurons, 9 mice). **D** The traces show representative responses of WT and Tg2576 neurons to short 10 pA-stepped depolarizing current injections (scale: 20 ms; 15 mV; 100 pA), scaled to show the AP threshold. The dashed lines show the threshold potential measured from the first AP elicited. The plots show the amount of injected current to generate the first AP (rheobase) and the threshold value for WT and Tg2576 neurons (WT: *n* = 10 neurons, 6 mice; Tg2576: *n* = 11 neurons, 7 mice; *Rheobase*: **p = 0.0039; *Threshold*: *p = 0.0248, both with Welch’s *t*-test). **E** Spontaneous firing recorded from PN and PBP neurons of the VTA (scale: 1 s; 0.2 mV), and respective plots showing instantaneous firing frequency (WT, PN: *n* = 16 neurons, 4 mice; Tg, PN: *n* = 28 neurons, 7 mice; WT, PBP: *n* = 16 neurons, 5 mice; Tg2576, PBP: *n* = 11 neurons, 5 mice; Welch’s *t*-test, *p = 0.037)

To better understand the basis of neuronal susceptibility to degeneration, we wanted to identify the characteristics of Tg2576 DA neurons in the VTA. Despite the overall high heterogeneity of these neurons in the electrophysiological and molecular features along the mediolateral axis [45–47], the small size of the PN nucleus concedes the characterization of a rather homogeneous DA neuron sub-population in the VTA with similar firing properties that differ significantly from the more conventional mesostriatal neurons [46]. Thus, we next focused on neurons in the PN to perform electrophysiological recordings at 3 months of age in WT and Tg2576 mice. Confocal analysis of biocytin-filled neurons confirmed the dopaminergic identity of the recorded cells (Fig. 4A).

As shown from the traces in Fig. 4B (depolarizing pulses) and Fig. 4C (hyperpolarizing pulses), the identified PN DA neurons exhibited non-conventional electrophysiological properties such as moderate afterhyperpolarization (AHP) during action-potential (AP) firing and small hyperpolarization-induced sag, corresponding respectively to low amplitude of currents mediated by Small-Conductance Calcium-Activated K^+^ (SK current) and HCN channels (I_h_ current) during voltage-clamp recordings (Table 1). Of note, these properties did not differ between WT and Tg2576 neurons (*AHP*: WT -56.6 ± 1.5 mV, Tg − 56.9 ± 2.0 mV; *p* = 0.9080; *sag*: WT -18.35 ± 5.32 mV, Tg − 19.98 ± 3.95 mV; *p* = 0.8076; *n* = 10–12 cells each). Yet, the DA neurons from Tg2576 mice showed a tendency to fire more AP during depolarization (Fig. 4B). This early feature in Tg2576 neurons did not appear to be due to changes in membrane resistance (Fig. 4C), or other AP properties (Table 1), but coincided with a significant reduction in the rheobase current – i.e. the minimal current needed to induce an AP – and, consequently, with a more hyperpolarized AP threshold (Fig. 4D). Indeed, DA neurons in Tg2576 mice required less current for AP firing, suggesting an increased neuronal excitability. Of note, in line with the tendency for increased excitability of PN Tg2576 neurons in response to current input, the spontaneous firing activity of putative DA neurons in the PN was also slightly, yet insignificantly, increased. Hyperexcitability was particularly pronounced in the neighboring PBP (Fig. 4E). The reduced rheobase and hyperpolarized AP threshold of 3-month-old PN DA neurons were accompanied by a reduction in cell capacitance (Cm; Table 1), suggesting a reduction in cell size in the transgenic animals. Interestingly, neuronal hyperexcitability and cell shrinking were also previously observed for DA neurons in the lateral VTA of 6-month-old mice [50], indicating that these changes are a common characteristic of VTA neurons in Tg2576 mice.

### Stereological analysis of VTA DA neuron subpopulations expressing Ca^2+^-binding proteins

The mitochondrial dysfunctions and the neurodegenerative process occurring in VTA DA neurons of Tg2576 mice likely correlate with deficits in the ability to correctly buffer intracellular Ca^2+^. Yet, mesolimbic DA neurons also express different Ca^2+^-binding proteins whose function in buffering cytoplasmatic Ca^2+^ was shown to protect neurons against mitochondria-affecting neurotoxins [91–96]. Thus, focusing on the VTA, we next analyzed the expression of CB and CR, two Ca^2+^-binding proteins expressed in midbrain DA neurons [97–100]. We used double-labelling immunofluorescence to visualize TH^+^ neurons expressing one of these Ca^2+^-binding proteins. Then, by stereological approach we quantified the dopaminergic neurons expressing CB (TH^+^CB^+^) or CR (TH^+^CR^+^) along aging.

**Fig. 5:**
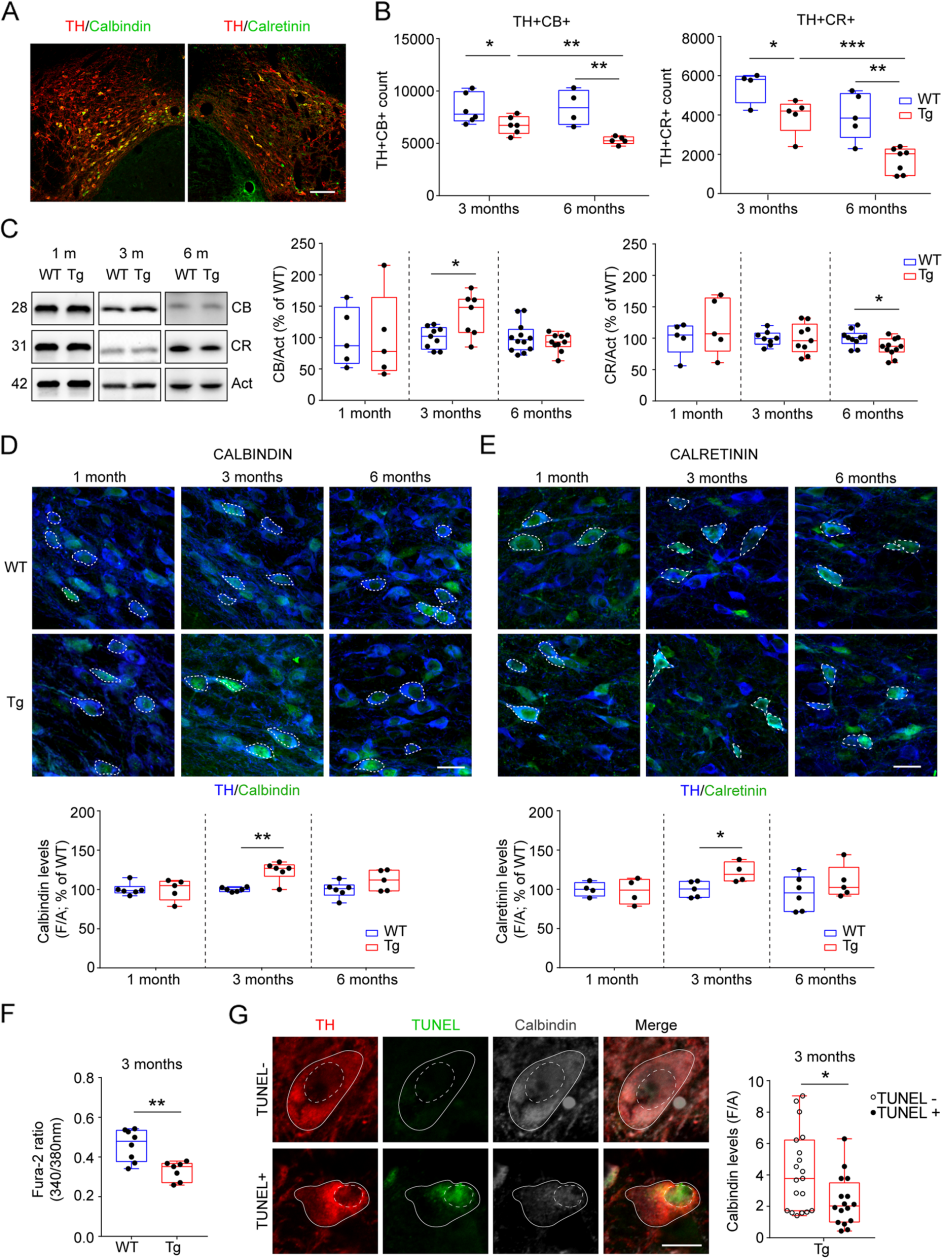
Overexpression of CB and CR in DA neurons of the Tg2576 VTA. **A.** Representative confocal image of coronal midbrain section showing TH^+^ (in red) together with Calbindin (CB^+^) or Calretinin (CR^+^) (both in green; scale: 100 μm). **B.** Stereological quantification of TH^+^ neurons co-expressing Ca^2+^-binding proteins (CB or CR) in the VTA of WT and Tg2576 mice of 3- and 6- months of age. TH^+^CB^+^ (*3 months*: WT: *n* = 6; Tg2576: *n* = 6; *6 months*: WT: *n* = 4; Tg2576: *n* = 5 mice. Two-way ANOVA for Genotype vs Age: Interaction: F_1,17_ = 2.343, *p* = 0.1443; Age: F_1,17_ = 1.625, *p* = 0.2196; Genotype: F_1,17_ = 20.72, *p* = 0.0003. WT 3 m vs Tg2576 3 m: Unpaired *t*-test: *p = 0.0447; WT 6 m vs Tg2576 6 m: Unpaired *t*-test: **p = 0.0048; Tg2576 3 m vs Tg2576 6 m: Unpaired *t*-test: **p = 0.0067). TH^+^CR^+^ (*3 months*: WT: *n* = 4; Tg2576: *n* = 5; *6 months*: WT: *n* = 5; Tg2576: *n* = 7 mice. Two-way ANOVA for Genotype vs Age: Interaction: F_1,17_ = 0.834, *p* = 0.3714; Genotype: F_1,17_ = 22.66, *p* = 0.0002; Age: F_1,17_ = 22.6, *p* = 0.0002; WT 3 m vs Tg2576 3 m: *p = 0.0352; WT 6 m vs Tg2576 6 m: **p = 0.0017; Tg2576 3 m vs Tg2576 6 m: ***p = 0.0005). **C.** Representative western blots from the midbrain of 1-, 3- and 6-month-old WT and Tg2576 mice, and plots showing the levels (expressed as % of WT) of CB (*left panel*) or CR (*right panel*) normalized to Actin as loading control. CB: 1 month: *n* = 5 mice per genotype; 3 months: WT: *n* = 9; Tg2576: *n* = 7 mice; Unpaired *t*-test: *p = 0.0156; 6-months: WT: *n* = 12; Tg2576: *n* = 10 mice. CR: 1 month: *n* = 5 mice per genotype; 3 months: WT: *n* = 8; Tg2576: *n* = 9; 6-months: WT: *n* = 10; Tg2576: *n* = 11; Unpaired *t*-test: *p = 0.0228. **D-E.** Panels show the analysis of confocal Z-stack double immunofluorescent labelling for TH (blue) and CB (**D**) or CR (**E**; both in green) in the VTA of coronal sections from 1-, 3- and 6-months-old WT and Tg2576 mice (scale: 20 μm). Box and whisker plots show the densitometric analysis of CB and CR levels in VTA TH^+^ neurons (**D.** 1 months-old: WT *n* = 6 mice, Tg2576 *n* = 5 mice; 3 months-old: *n* = 6 mice per genotype; Welch’s: **p = 0.0046; 6 months-old: WT *n* = 6 mice, Tg2576 *n* = 5 mice. **E.** 1 months-old: *n* = 4 mice per genotype; 3 months-old: WT *n* = 5 mice, Tg2576 *n* = 4 mice; Unpaired *t*-test: *p = 0. 0263. 6 months-old: WT *n* = 6 mice, Tg2576 *n* = 5 mice). **F.** The plot shows the amount of free [Ca^2+^] expressed as 340/380 ratio of Fura-2 in the PN o 3-month-old WT and Tg2576 mice (WT: *n* = 9 neurons,3 mice; Tg2576: *n* = 10 neurons, 3 mice; Welch’s *t*-test, **p = 0.0024). **G.** Representative confocal images of TUNEL-negative (TUNEL^-^) and -positive (TUNEL^+^) dopaminergic neurons expressing CB from the VTA of 3-month-old Tg2576 mice (scale bar: 10 μm). The graph shows densitometric levels of CB in TUNEL^-^ and TUNEL^+^ neurons (TUNEL^-^: *n* = 19 neurons; 3 mice; TUNEL^+^: *n* = 16 neurons; 3 mice; Unpaired *t*-test: *p = 0.0141)

As shown in Fig. 5A, both CB and CR are expressed in DA neurons in the VTA. Importantly, already at 3 months of age we could observe a significant reduction in the number of TH^+^CB^+^ or TH^+^CR^+^ neurons in Tg2576 compared to WT mice, which became even stronger at 6 months of age. Additionally, although WT mice showed constant neuron numbers with age, Tg2576 mice showed progressive loss of neurons expressing these proteins from 3 to 6 months of age (Fig. 5B), suggesting an age-dependent degeneration.

### VTA dopaminergic neurons in Tg2576 mice overexpress Ca^2+^-binding proteins

The loss of neurons expressing Ca^2+^-binding proteins in Tg2576 mice was striking, as we had expected that the presence of these proteins would make DA neurons less vulnerable to cell death. To investigate this further, we focused on evaluating the expression levels of both CB and CR in the entire midbrain from WT and Tg2576 mice by western blot. We readily observed higher expression levels of CB in the midbrain of 3-month-old Tg2576 mice compared to WT, while no changes were observed for CR levels at this age (Fig. 5C). Interestingly, the levels of CB in 6-month-old Tg2576 mice were equal to those of WT mice. No changes were observed prior to the beginning of neurodegeneration (at 1 month of age; Fig. 5C). Thus, the onset of neurodegeneration at 3 months of age coincides with CB overexpression. On the other hand, at 6 months of age the CR levels are lower in the midbrain of Tg2576 mice compared to WT. Yet, the fact that CR protein levels are only slightly lower but are accompanied by a much stronger reduction in the number of CR^+^ neurons in Tg2576 mice (see Fig. 5B), would suggest that also this protein is overexpressed in the Tg2576 midbrain.

To verify this hypothesis and focus solely in the VTA, we analyzed in more detail the expression levels of CB and CR in DA neurons by confocal microscopy. At 3 months of age, we observed a significant increase in the levels of both CB and CR in TH^+^ neurons from Tg2576 mice compared to WT cells, whereas the levels of these proteins remained slightly – yet insignificantly – higher at 6 months of age (Fig. 5D,E). Instead, at 1 month of age, prior to the insurgence of neurodegenerative events, CB and CR levels in TH^+^ neurons are similar between genotypes.

Given the overexpression of Ca^2+^-binding proteins in 3-month-old DA neurons of Tg2576 mice, we next hypothesized that somatic cytoplasmic Ca^2+^ levels would be decreased. To verify this hypothesis, we performed Ca^2+^-microfluorometry measurements from PN neurons during patch-clamp recordings from acute midbrain slices. As expected, we observed significantly lower Ca^2+^ levels in neurons from Tg2576 mice (Fig. 5F). Overall, these data could suggest that DA neurons in Tg2576 mice start to overexpress CB or CR at the onset of degeneration at 3 months of age, likely as a neuroprotective measure against Ca^2+^-induced toxicity.

Finally, to further investigate the role of Ca^2+^-binding proteins in association with more resistance to degeneration at this age, we analyzed CB levels in VTA dopaminergic neurons displaying normal or pyknotic nuclei. Dying neurons, in fact, show an apoptotic profile with chromatin condensation that can be revealed by TUNEL assay. Interestingly, we detected higher CB levels in dopaminergic neurons negative for TUNEL assay compared to TUNEL-positive ones (Fig. 5G). To exclude that the decreased levels of CB in dying dopaminergic neurons was due to a general block of translational events, we additionally measured the TH levels in Tg2576 neurons, and found them unaltered between TUNEL-positive and -negatives cells (Suppl. Fig. 1D). These data indicate that the absence of apoptotic features in Tg2576 neurons is associated with high levels of CB.

## Discussion

In this study, we report that the degenerative process involves DA neurons in the PN and PBP subnuclei of the VTA, that project to the NAc core and medial shell, leading to reduction of DA levels in the ventral striatum of Tg2576 mice. The degeneration appears to be mediated by the accumulation of abnormal mitochondria, and by translocation of AIF from the mitochondrial inner membrane to the nucleus. Of note, at the onset of degeneration (3 months of age), the DA neurons in Tg2576 mice show increased neuronal excitability. Importantly, they also show overexpression of Ca^2+^-binding proteins, accompanied by lower free Ca^2+^ levels, that – we can assume – act as neuroprotective mechanisms, attempting to contribute to neuronal survival during the disease progression.

The present work confirms our earlier observation [21] that the loss of DA primarily involves mesocorticolimbic brain areas. Indeed, we saw here that the degeneration is selective for neurons in the VTA and is translated in a strong reduction of evoked DA and dopaminergic innervation in the NAc core and medial shell at 4 months of age. Moreover, our retrograde experiments confirmed that these brain regions are innervated exclusively by dopaminergic neurons in the PN and PBP, in line with earlier observations from different labs [46, 84–86]. Conversely, the dorsal striatum of Tg2576 mice – innervated by SNpc DA neurons – shows normal levels of evoked DA and normal dopaminergic fiber staining. This is in line with the fact that SNpc DA neuron numbers are unaffected in Tg2576 mice. The reasons for the selective vulnerability of VTA over SNpc DA neurons in AD is still unknown and can be the basis of intense work in the future. Of note, these neurons have specific intrinsic characteristics that differentiate them from each other and might be the key to the different pathophysiological responses of the VTA *vs* SNpc cells to metabolic, genetic, environmental or lifestyle risk factors.

Given the well-known relationship between the dopaminergic VTA and the reward system [101, 102], the loss of mesocorticolimbic DA fits well with the neuropsychiatric-like symptoms we previously observed in Tg2576 animals, evident as an impairment in reward processing when tested in the chocolate-elicited conditioned place preference task [21]. It is also in line with other non-cognitive and memory-related deficits in these mice [21, 38, 39, 103, 104], in view of the fact that the degeneration in the PN and PBP also affects neurons projecting to areas other than the NAc. Importantly, our data provide an intriguing explanation for the early manifestation of neuropsychiatric symptoms observed in clinical AD [3–5, 105, 106], particularly depression and apathy, associated with strong functional disconnection of the VTA with the default-mode network since the MCI stage [22, 23].

Here, we demonstrate an accumulation of swollen and vacuolated mitochondria in the cytoplasm of VTA dopaminergic neurons of Tg2576 mice, a clear sign of cell distress. This is in strict compliance with our earlier observations of deficits in the autophagic flux, increased accumulation of autophagosomes and of c-Abl activation (a tyrosine kinase involved in neurodegenerative diseases) in 3-month-old DA neurons, at the onset of degeneration [50]. Taken together, these observations might suggest that the process of mitophagy – the selective autophagic turnover of damaged mitochondria [107] – is dysfunctional in VTA dopaminergic neurons, contributing to Ca^2+^-mediated neurotoxicity. Indeed, Ca^2+^ overload due to mitochondria dysfunction, inhibition of respiratory complexes and enhanced ROS production is known to be a key player for cell death [53].

Of note, it has been demonstrated that a block of autophagy flux could correlate with the induction of the AIF-mediated apoptosis [88], a mechanism that seems implicated in AD [57, 59, 60] and in degeneration events affecting the SNpc dopaminergic neurons in PD mouse models [55, 56, 58]. Thus, here we investigated the role of AIF in VTA neuronal loss in our 3-month-old mice. Interestingly, we detected translocation of AIF from the mitochondria to the nucleus of Tg2576 neurons, where it can induce chromatin condensation, chromatolysis and caspase-independent cell death [55, 56, 58, 89, 108]. The cleavage of AIF from the mitochondrial inner membrane of Tg2576 neurons is likely mediated by calpain, a Ca^2+^-sensitive protease that is activated when Ca^2+^ levels in the cytoplasm are abnormally increased [90, 109]. In line with this notion, calpain levels were shown to be elevated in post-mortem PD tissues [110], whereas inhibition of calpain can prevent the loss of SNpc DA neurons following mitochondria-damaging toxins like MPTP [111].

One of the most striking results of our experiments was the observation that, during the progression of DA neuron degeneration in Tg2576 mice, also the neurons expressing Ca^2+^-binding proteins were affected, in an age-dependent manner. This is analogous to data from other studies showing progressive loss of other vulnerable neuronal populations expressing Ca^2+^-binding proteins in AD, such as CB-containing cholinergic neurons of the basal forebrain or CR- and parvalbumin-positive neurons in the hippocampus [112, 113]. At first glance our data were intriguing, given the many works from patients and animal models of PD showing that dopaminergic neurons containing Ca^2+^-binding-proteins, particularly CB, are relatively preserved from neurodegeneration both in the SNpc and VTA [95, 96, 107, 114–116], suggesting that CB has a neuroprotective role, particularly against mitochondria-damaging toxins like MPTP [94–96, 99, 117–120]. Indeed, Ca^2+^-binding proteins in neurons regulate the availability of free Ca^2+^ in the cytoplasm, and their role can become essential for neuroprotection, particularly when the mitochondria fail to buffer Ca^2+^.

Thus, given our observations of mitochondrial dysfunction in the Tg2576 DA neurons, and the eventual need for Ca^2+^ buffering by Ca^2+^-binding proteins, we investigated not only the *presence* per se of CB and CR in our cells, but also their *levels* in TH^+^ neurons, to understand how these are altered during the disease progression in Tg2576 mice. We observed higher levels of both CB and CR in Tg2576 neurons compared to WT ones at the early degeneration phase (3 months of age). Our data are in line with a previous work showing that mitochondrial insult by MPTP can induce an overexpression of CB in midbrain neurons and an increase in the numbers of CB-containing cells [94]. Overexpression of Ca^2+^-binding proteins, including CB, calmodulin and calregulin, has also been observed in post-mortem TH^+^ neurons from PD patients [93]. Moreover, overexpression of CB in transgenic animals was shown to confer neuroprotection and enhanced DA levels against MPTP toxicity [96]. Importantly, our data from AD mice are in line with the notion that an overall modulation of the Ca^2+^ machinery (i.e regulation of Ca^2+^-binding protein levels, Ca^2+^ channels, pumps or exchangers) in stressed dopaminergic neurons can be neuroprotective during mitochondrial insults (see for examples [121] for neuroprotection by the L-type Ca^2+^ channel blocker isradipine, [122] for neuroprotection by glutamatergic receptor antagonists; and also [123, 124]). Accordingly, we observed lower levels of cytoplasmic Ca^2+^ in Tg2576 dopaminergic neurons during our electrophysiological recordings, presumably as result of Ca^2+^-binding protein upregulation. Indeed, the levels of CB inversely correlate with cell death in 3-month-old Tg2576 mice, where we observed lower levels of CB in TUNEL-positive dopaminergic neurons. The fact that TUNEL^+^ neurons show unaltered TH levels might confirm that reduction of CB is not a generic effect of protein synthesis failure but is a specific event linked to increased susceptibility. These data lead us to interpret that CB is associated with cell surviving, at least at the examined age.

Of note, other than alterations in the content of CB or CR, the PN neurons in Tg2576 mice also showed alterations in electrophysiological properties such as enhanced neuronal firing and more depolarised AP threshold (see also [50]). The mechanisms underlying these changes are still not clear, but they are likely the result of different molecular changes occurring in these neurons. An intriguing hypothesis is that the overexpression of CB leads to neuronal hyperexcitability to compensate for the reduced probability of DA release in projection areas, given the inverse relationship between CB levels and synaptic exocytosis [91, 125]. It is also interesting to note that the DA neurons we were able to record from the VTA of 3-month-old Tg2576 mice are those with unconventional properties like small I_h_ current (see Table 1). Curiously, low I_h_-containing DA neurons were shown to be resistant to degeneration in a *Drp1* knockout mouse, despite almost complete loss of axonal mitochondria, and are the same neurons that mostly survive mitochondria insults [126], raising the hypothesis that low I_h_ can be considered a marker of ‘resistant’ neurons. Incidentally, these low I_h_-containing DA neurons have been shown to be the neurons with higher expression of CB in the medial VTA [127].

## Conclusions

In summary, the degeneration of DA neurons in the VTA of AD mice mainly affects those neurons projecting to the NAc and it involves defects in mitochondria and alterations in the homeostasis of Ca^2+^. Conversely, the DA neurons that were alive in 3-month-old Tg2576 mice overexpress Ca^2+^-binding proteins, which likely confer these neurons some degree of neuroprotection against cell death. Unfortunately, we are unable to affirm if neurons with Ca^2+^-binding protein overexpression will be protected enough to be able to survive during the disease progression at more advances ages. Understanding the basis of the selective resistance of a given dopaminergic neuron subpopulation is particularly important and relevant for both AD and PD. Our results of Ca^2+^-binding protein upregulation go in the right direction to discover why vulnerable neurons degenerate and which are the mechanisms underlying mitochondrial dysfunction. Our data can be particularly relevant also in light of evidence showing that antioxidant diet supplements, such as high levels of vitamin A (and the derivative retinoic acid), regulate the expression of Ca^2+^-binding proteins [128] and improve memory and cognition in AD mice and patients [129–132]. Thus, clinical and nutritional approaches focusing on the regulation of Ca^2+^ homeostasis in the VTA can be a promising adjuvant treatment for AD prevention.

## Supplementary Information

**Additional file 1: Fig. S1.** Additional data from 3-months old mice. A. Evoked DA concentration in NAc shell, NAc core and dorsal striatum (WT: *n* = 21–32 observations from 9 to 12 slices, 4 mice; Tg2576: *n* = 18–30 observations from 9 to 11 slices, 4 mice) and example amperometric traces from 3-month-old WT and Tg2576 mice (scale: 100 pA) recorded with a carbon fiber electrode of equal calibration. B. Representative immunofluorescent labelling (scale bar, 200 μm) for TH in 3-month-old WT and Tg2576 mice and graphs showing densitometric values of TH levels in projecting areas. *Left panels*: NAc coronal sections showing the NAc shell (asterisk; *n* = 5 per genotype) and NAc core (arrowhead; *n* = 5 per genotype). *Right panels*: dorsal striatum (scale bar, 50 μm. *n* = 5 per genotype). C. As in B, but showing immunofluorescent labelling (scale bar, 200 μm) for DAT in NAc shell (asterisk; *n* = 5 per genotype), NAc core (arrowhead; *n* = 5 per genotype) and dorsal striatum (scale bar, 50 μm. *n* = 5 per genotype). D. The graph shows densitometric levels of TH in TUNEL^-^ and TUNEL^+^ neurons (TUNEL^-^: *n* = 19 neurons; 3 mice; TUNEL^+^: *n* = 16 neurons; 3 mice) from the VTA of 3-month-old Tg2576 mice.

## Data Availability

All data generated or analyzed during this study are included in this article and its supplementary information files.

## Abbreviations

Aβ: Amyloid beta
AD: Alzheimer’s Disease
AHP: After hyper polarization
AIF: Apoptosis-Inducing Factor
AP: Action-potential
Ca^2+^: Calcium
CB: Calbindin-D28K
Cm: Cell capacitance
CR: Calretinin
DA: Dopamine
DAT: Dopamine transporter
MCI: Mild cognitive impairment
NAc: Nucleus accumbens
PBP: Parabrachial pigmented nucleus
PD: Parkinson’s Disease
PN: Paranigral nucleus
ROS: Reactive oxygen species
SN*pc*: Substantia Nigra *pars compacta*
TEM: Transmission electron microscopy
TH: Tyrosine hydroxylase
VTA: Ventral Tegmental Area
WT: Wild-type
