# Supplementary figures and images for: Upregulation of Ca2+-binding proteins contributes to VTA dopamine neuron survival in the early phases of Alzheimer’s disease in Tg2576 mice
Source: Mol Neurodegener. 2022 Nov 25;17:76. doi: 10.1186/s13024-022-00580-6 (PMC9700939; doi:10.1186/s13024-022-00580-6)

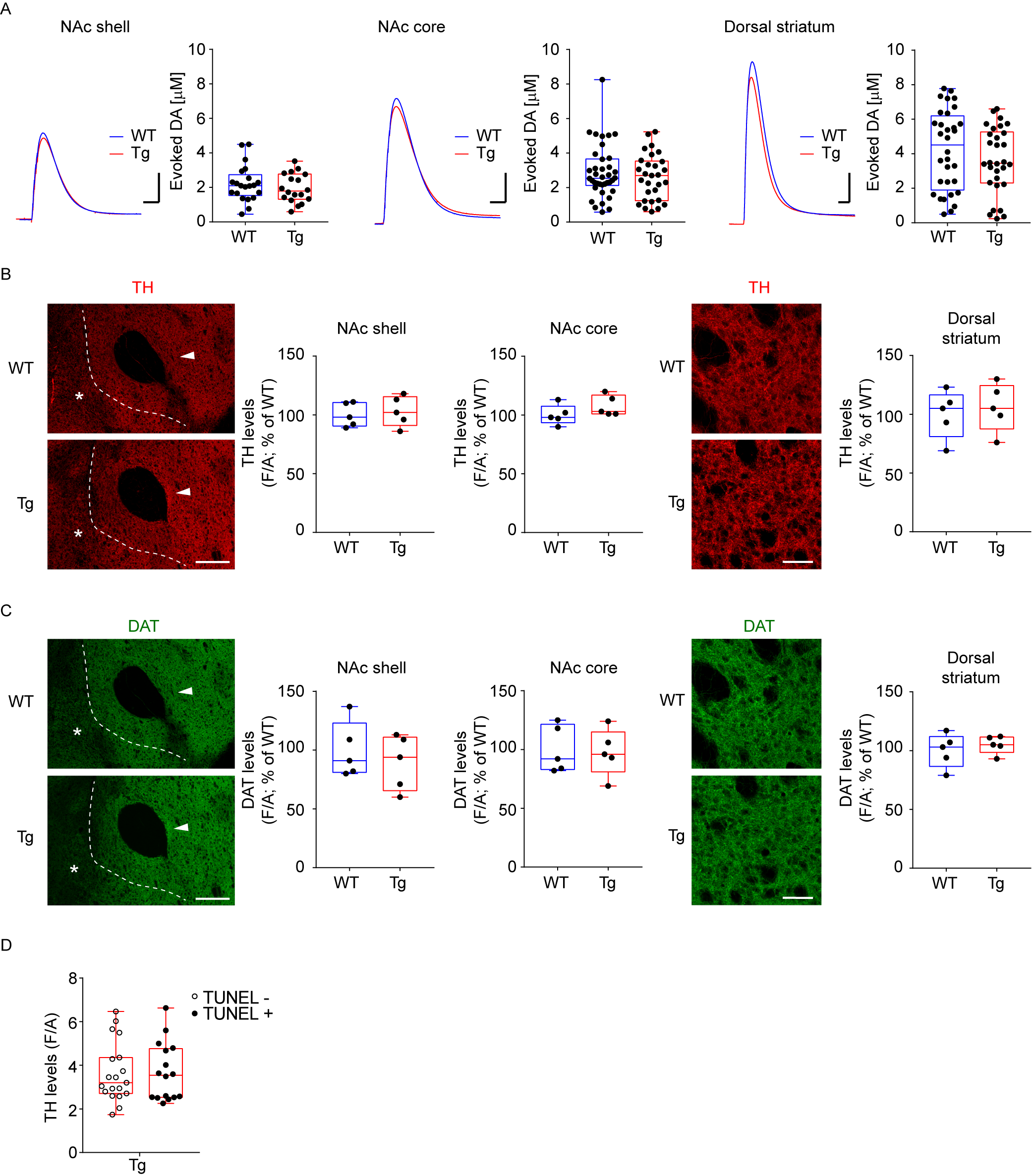

Supplement: Supplementary file 1 — Additional file 1: Fig. S1. Additional data from 3-months old mice. A. Evoked DA concentration in NAc shell, NAc core and dorsal striatum (WT: n = 21–32 observations from 9 to 12 slices, 4 mice; Tg2576: n = 18–30 observations from 9 to 11 slices, 4 mice) and example amperometric traces from 3-month-old WT and Tg2576 mice (scale: 100 pA) recorded with a carbon fiber electrode of equal calibration. B. Representative immunofluorescent labelling (scale bar, 200 μm) for TH in 3-month-old WT and Tg2576 mice and graphs showing densitometric values of TH levels in projecting areas. Left panels: NAc coronal sections showing the NAc shell (asterisk; n = 5 per genotype) and NAc core (arrowhead; n = 5 per genotype). Right panels: dorsal striatum (scale bar, 50 μm. n = 5 per genotype). C. As in B, but showing immunofluorescent labelling (scale bar, 200 μm) for DAT in NAc shell (asterisk; n = 5 per genotype), NAc core (arrowhead; n = 5 per genotype) and dorsal striatum (scale bar, 50 μm. n = 5 per genotype). D. The graph shows densitometric levels of TH in TUNEL- and TUNEL+ neurons (TUNEL-: n = 19 neurons; 3 mice; TUNEL+: n = 16 neurons; 3 mice) from the VTA of 3-month-old Tg2576 mice. [file 13024_2022_580_MOESM1_ESM.tif]
